# Supplementary material for: Cultural and Medicinal Use of Amphibians and Reptiles by Indigenous People in Punjab, Pakistan with Comments on Conservation Implications for Herpetofauna
Source: Animals (Basel). 2022 Aug 13;12(16):2062. doi: 10.3390/ani12162062 (PMC9405124; doi:10.3390/ani12162062)
Supplement: Supplementary file 1 [file animals-12-02062-s001.zip › animals-1791709-supplementary.pdf]

RESEARCH CONSENT FORM

CONSENT

I have read, and I understand the provided information and have had the ability to give answers to the following questions. I understand that my participation is voluntary. I understand that I will be given a copy of this consent form. I voluntarily agree to take part in this study.

Researchers are bound by a *code of ethics* that includes the following protections

1. *Protection of privacy and confidentiality*
2. *Protection against unjustifiable deception*

Informant's Sign ______________________________ Date __________

Researcher's Sign______________________________ Date __________

Table S1. Ethno-biological questionnaire form

Enumerator	Respondent name
Village name	Ethnic background
Occupation	Age
Which species have you seen?	
---<br>---<br>----------	
Local name of the species	
---<br>---<br>----------	
Uses of species as food	
---<br>---<br>----------	
Uses of species as medicine	
---<br>---<br>----------	
Superstitious about species	
---<br>---<br>----------	
Uses of species as other purposes	
---<br>---	

Important note

Table S2. Inclusion and exclusion criteria for the survey

**Included**	**Excluded**
survey conducted at individual level	survey collected at group i.e., schools, universities, colleges
surveys conducted with local peoples	surveys conducted with foreigners
≥ 18 ages	< 18 ages
survey conducted at regional level	survey collected at national level
